# Integrative analyses of translatome and transcriptome reveal important translational controls in brown and white adipose regulated by microRNAs

**DOI:** 10.1038/s41598-017-06077-3

**Published:** 2017-07-18

**Authors:** David W. Reid, Dan Xu, Peng Chen, Hongyuan Yang, Lei Sun

**Affiliations:** 1 Cardiovascular and Metabolic Disorders Program, Duke-NUS Graduate Medical School, 8 College Road, Singapore, 169857 Singapore; 2 Institute of Molecular and Cell Biology, 61 Biopolis Drive, Proteos, Singapore, 138673 Singapore; 3 School of Biotechnology and Biomolecular Sciences, University of New South Wales, Sydney, 2052 Australia; 4 School of Laboratory Medicine and Life Science, Wenzhou Medical University, Wenzhou, Zhejiang 325035 China; 5 Division of Bioengineering, Nanyang Technological University, 70 Nanyang Drive, Singapore, 637457 Singapore

## Abstract

The epidemic of obesity and diabetes has markedly spurred the research interest in adipocyte biology. Brown adipocytes are specialized for energy expenditure and of therapeutic interest for treatment of metabolic diseases, but how brown adipocytes are distinguished from white adipocytes at the level of translational regulation remains poorly understood. To systemically determine the translational control of gene expression in adipose tissue, we performed ribosome profiling and RNA-seq in parallel to depict the translatome and transcriptome changes during primary brown and white adipogenesis, and between brown and white adipose tissue. The most prominent layer of translational regulation was the increased translation efficiency of genes encoding mitochondria components in brown adipocytes relative to white. Systemic analysis of the regulatory interactions between microRNAs and their targets revealed that microRNAs were more active in repressing targets’ mRNA abundance and translation in brown fat. Together, our data comprehensively delineated a landscape integrating transcriptome and translatome in adipose tissue.

## Introduction

As the worldwide incidence of obesity and its associated diseases has increased^1, 2^, the past decade has seen a rapid growth of interest in adipose biology. There are at least three major types of adipose tissue in mammals: white, brown and beige. While white adipose tissue ﻿(WAT)﻿ is distributed throughout our bodies and store excessive energy in the form of triglyceride (TAG), brown adipose tissue﻿ (BAT), found in the interscapular region in rodents and human infants, can burn stored lipids to generate heat as a defense against cold temperatures and obesity^3–6^. Beige adipocytes mainly exist in the subcutaneous white adipose tissue. They manifest features of white adipocytes at thermoneutrality, but take on thermogenic gene expression program in response to cold exposure or β-adrenergic receptor agonists^3, 5, 7^.

A large number of studies have been conducted to determine the detailed molecular mechanism underlying adipogenesis and lineage-specific features^5, 6, 8–13^. Epigenetic and posttranscriptional controls of gene expression are emerging as new and important elements in the regulatory network governing adipocyte biology. A comparative epigenomic analysis by ChIP-seq revealed an extensive chromatin remodeling during adipocyte differentiation^14^. DNA methylation contributes to the lineage specific development of brown and white adipocytes^15^. Recent studies from our and other groups have demonstrated the functional involvement of long non-coding RNAs in adipocyte differentiation and lineage determination by modulating the activity of chromatin-modifying complex^13, 16–18^. A polysome profiling study revealed extensive posttranscriptional regulation during the initial steps of human adipocyte stem cell differentiation^19^. Dai *et al*. showed that IMP2, a RNA binding protein, could directly bind and inhibit Ucp1 mRNA translation, and Igf2bp2/Imp2 knockout mice developed resistance to obesity due to enhanced translation of Ucp1^20^, which illustrates an example of translational control for some key lineage-specific factors. Despite this progress, the genome-wide translational control during lineage-specific development has yet to be determined.

MicroRNAs are essential regulators for adipocytes^21–24^. Adipose-specific knockout of Dicer or Dgcr8, two essential proteins for microRNA biogenesis, results in depletion of most microRNAs, leading to a WAT-like phenotype in brown adipose tissue (BAT) and a defect in WAT development^25, 26^; even heterozygous knockout of Dicer in BAT can decrease the expression of Ucp1 and aggravate obesity-evoked deterioration of glucose metabolism^27^. MicroRNA can regulate gene expression through mRNA destabilization and translational repression, however, the respective influence of microRNAs on these two aspects in adipose tissue has not been explored.

To provide an integrative view of translatome and transcriptome in brown and white fat, we conducted ribosome profiling and RNA-seq for primary brown and white adipocyte cultures, and brown and white adipose tissue. We found that the translation of genes encoding mitochondria components is enhanced more during brown adipogenesis than during white adipogenesis, and higher in brown than white adipose. Moreover, we revealed a differential contribution of microRNAs to translational repression in BAT and WAT. Together, these data provide a framework to study the translational regulation in adipose lineage-specific development.

## Results and Discussion

### Ribosome profiling of adipocytes

**Figure 1:**
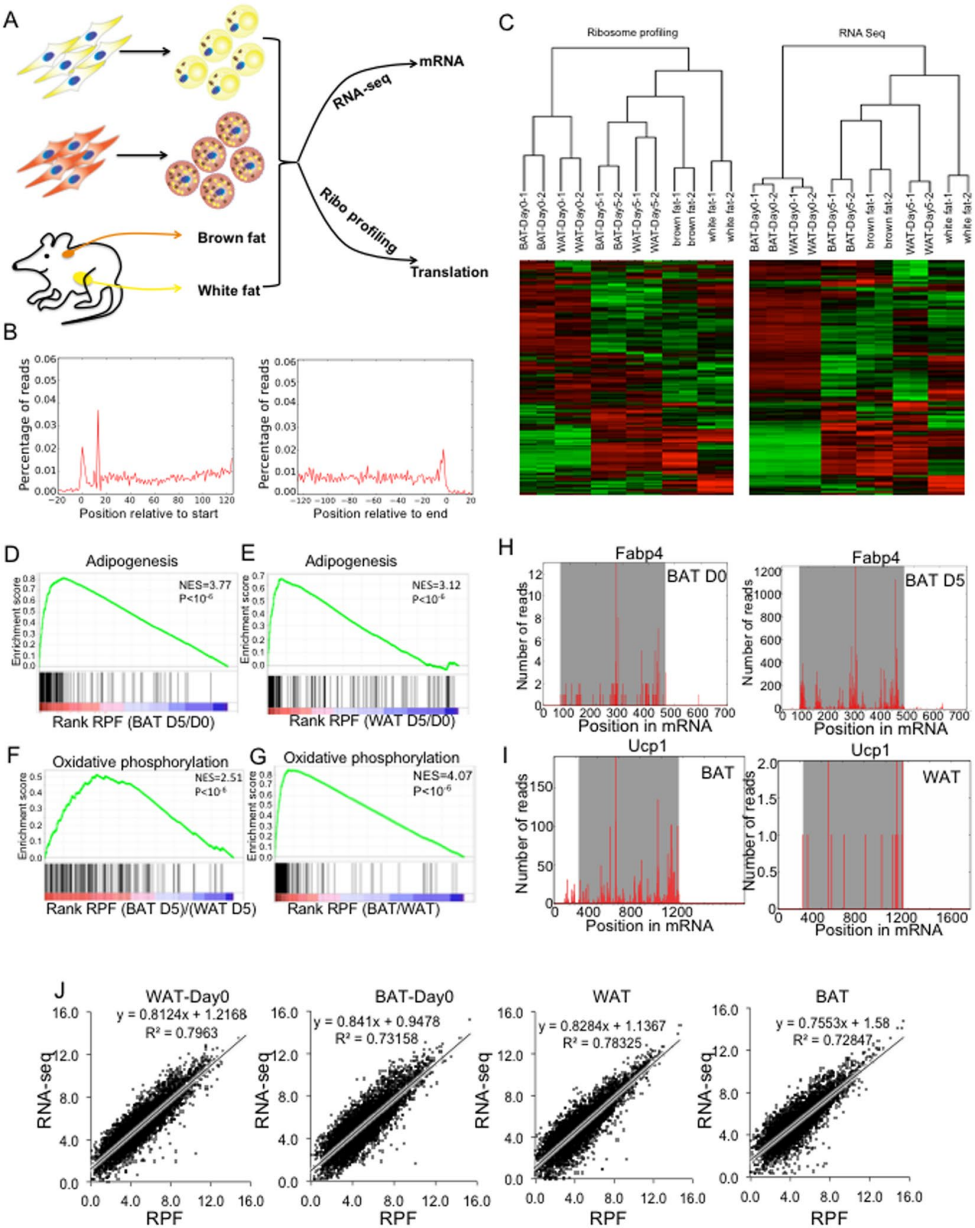
Ribosome profiling in adipocytes. (**A**) Schematic diagram of the experimental design. Primary brown and white preadipocytes were isolated from interscapular brown adipose (BAT) and inguinal white adipose (WAT), cultured *in vitro* for differentiation. Cells at day 0 and day 5 were harvested for RNA-seq and ribosome profiling in parallel. BAT and epididymal white adipose tissue (eWAT) were harvested for RNA-seq and ribosome profiling. (**B**) Metagene analysis of translation initiation and termination. Average ribosome footprint density profiles of all the detectable mRNAs in BAT are aligned at their start codon and stop codon. (**C**) Hierarchical clustering of all samples in this studies based on their mRNA expression and RPF. (**D**–**G**) GSEA analysis of ribosome profiling data during (**D**) brown and (**E**) white adipogenesis (D5/D0); (**F**) Cultured brown adipocyte vs. white adipocyte (BAT D5)/(WAT D5); (**G**) BAT vs WAT (BAT/WAT). (**H**) Examples of ribosome profiling data over two genes: Fabp4 in brown adipocyte culture Day 0 vs. Day 5; (**I**) Ucp1 in BAT vs WAT. (**J**) Correlation between RNA-seq and ribosome profiling data in WAT-Day0, BAT-Day0, WAT and BAT samples.

In this study, we aimed to depict an integrative view of translatome by ribosome profiling and transcriptome by RNA-seq during adipocyte differentiation and between brown and white adipose tissue (BAT and WAT). To begin with, we isolated primary brown and white preadipocytes from mouse interscapular BAT and inguinal WAT (2–3 weeks old mice) and *in vitro* differentiation as described before^13, 28^. We performed Oil-red-O staining and real-time PCR to characterize the lipid accumulation and adipogenic marker expression (Fabp4 and Pparg). At day 5 of differentiation, lipid accumulation reached the peak and did not significantly increase after day 5 (Fig. 1SA). Similarly, the expression of Fabp4 and Pparg increased to the maximum at day 5 (Fig. 1SB). Importantly, both lipid accumulation and pan-adipogenic marker expression didn’t differ significantly at day 5 in brown and white adipocytes, indicating that they have the similar extent of differentiation. Thus, we harvested brown and white preadipocytes prior to induction (Day 0) and mature adipocytes at day 5 after induction for ribosome profiling^29^ and RNA-seq in parallel. In addition, interscapular BAT and epididymal WAT were directly harvested from 6-weeks animals for profiling (Fig. 1A). Each of these samples generated >10 million reads, which were mapped to annotated mRNAs for quantification. 5608 genes were covered by at least 10 raw reads in both ribosome profiling and RNA-seq and were considered for downstream analysis.

Combining ribosome protected fragments (RPF) from all mRNAs into one metagene show that majority of RPFs were mapped to annotated open read frames (ORF)s (Fig. 1B), reflecting the fundamental feature of mRNA translation. The density of RPF was highest at the start and stop codons (Fig. 1B), due to the pauses of ribosome at these positions^30^. In contrast, the mRNA-seq reads were uniformly mapped across the full length of mRNAs including 5′UTR and 3′UTR (not shown). 3-nucleotide periodicity could be observed in RPF but not the RNA-seq data. Thus, our ribosome profiling data accurately portray translational status.

To assess if mRNA and RPF carry similar molecular signature linked to cell type, we performed unsupervised hierarchical clustering separately on mRNA and RFPs. Duplicates of each sample were clustered together (Fig. 1C), indicating the strong correlation between our duplicates (Figure S2A,S2B). Two major branches separated preadipocytes from Day 5 mature adipocytes as well as adipose tissues, manifesting a large difference in the gene expression profile between preadipocytes and mature adipocytes. The dendrograms of RNA-seq data and ribosome profiling data by and large mirrored each other, indicating that both sets of data can reflect cell type-specific features.

Next, we calculated the correlation between samples in a pairwise comparison according to RPF or RNA, and plotted the correlation in a heatmap. Consistent with the dendrogram, preadipocyte samples showed strong correlation between each other regardless lineage, while differentiated cells and adipose tissues correlated well regardless their origin (Figure S2F,G). Similar patterns were observed for RPF and RNA levels.

To determine whether RPFs can reflect biological changes during adipogenesis and between distinct depots, we performed gene set enrichment analysis (GSEA) on pre-ranked gene list. The most up-regulated GO between D5 and D0 is adipogenesis during both BAT and WAT adipogenesis at RPF (Fig. 1D,E) as well as RNA levels (Figure S1C,D). For instance, a dramatic up-regulation was observed for several adipogenic markers such as Fabp4, Lipe, Acsl1, Pparg, Glut4, etc (Fig. 1H, Figure S3A). The most enriched GO in BAT relative to WAT depots as well as between cultured mature brown and white adipocytes was oxidative phosphorylation, a hallmark of BAT due to its abundant mitochondria (Fig. 1F,G and Figure S2E). For example, the RPF of Ucp1 was much higher in BAT than in WAT (Fig. 1I).

To examine the relationship between RNA and RPFs within each sample, we plotted the RNA-seq data against the RPF and found that they correlated tightly with R^2^ ranging from 0.7 to 0.8 (Fig. 1J), suggesting that RNA abundance was the major contributor to the total translation represented by RPFs.

Although the number of replicates (two) used in this study is limited, the correlation between our duplicates is high (Figure S2A,B). GO terms from distinct cell types differed considerably (Figure S2E), reflecting the different cell phenotypes. Thus, these high throughput data should be sufficient for us to perform downstream analysis.

### Translational control contributes more to RPF changes during brown than during white adipogenesis

**Figure 2:**
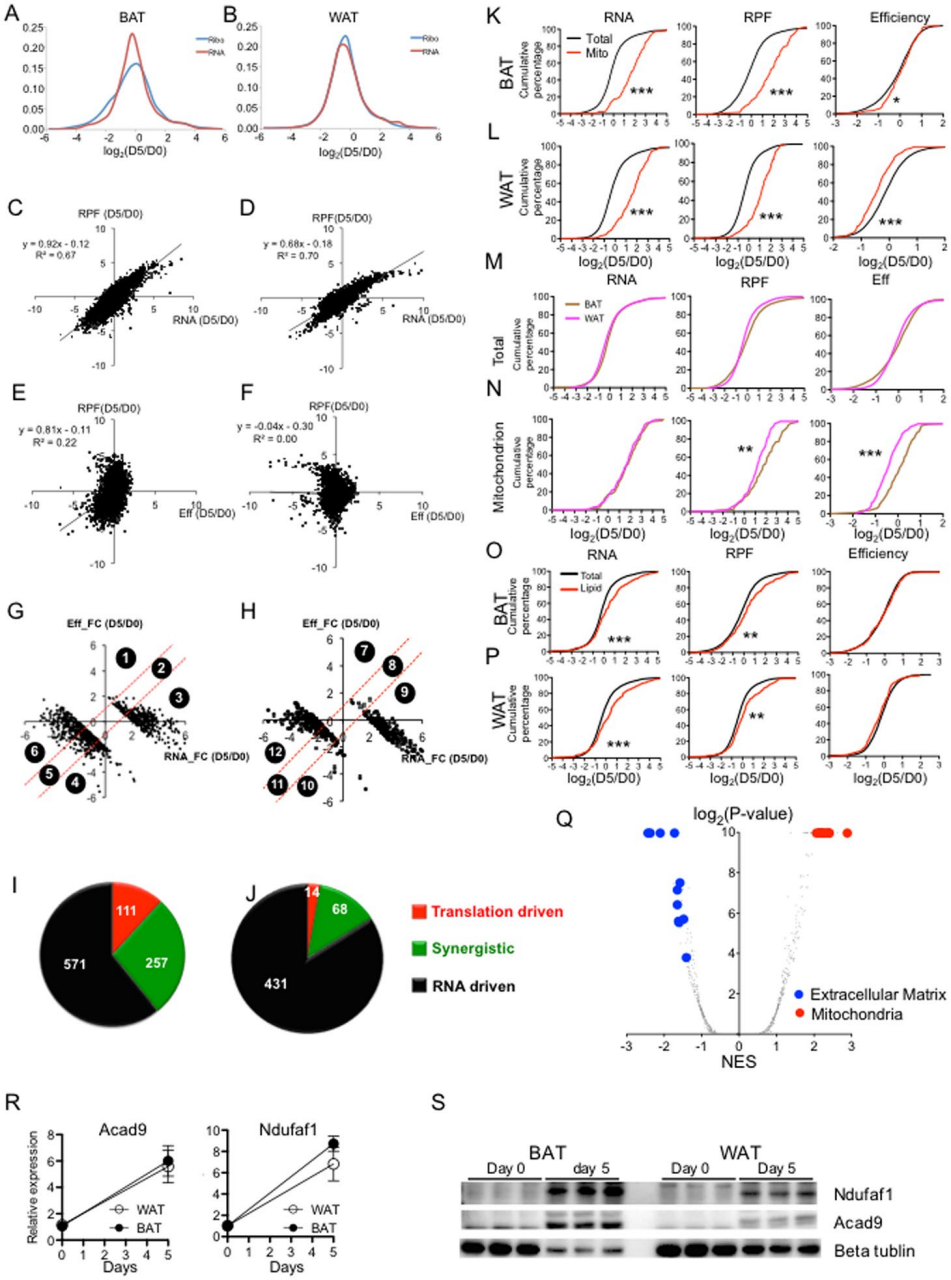
Genes encoding mitochondria components are more actively translated during brown adipogenesis than during white adipogenesis. (**A**,**B**) The distribution of RPF and RNA fold changes (FC) of each gene during (**A**) brown and (**B**) white adipocyte adipogenesis. (**C**,**D**) The correlation between RPF FCs and RNA FCs during (**C**) brown and (**D**) white adipogenesis. (**E**,**F**) The correlation between RPF FCs and translational efficiency (EFF) FCs during (**E**) brown and (**F**) white adipogenesis. (**G**,**H**) Based on the relative contribution of mRNA vs Eff FCs to the total translation FCs, 959 and 513 genes with ≥4 fold RPF changes in (**G**) brown and (**H**) white adipogenesis are classified into 6 groups. Genes with increased and decreased RFP comprise Group 1–3 and Group 4–6, respectively. (**I**,**J**) Pie charts to show the composition of each group during (**I**) brown and (**J**) white adipogenesis. Genes were classified into three types: translation driven, synergistic (driven by both RNA and translation), and RNA driven. (**K**,**L**) The cumulative fraction curves of (left) RNA-, (middle) RPF- and (right) Efficiency-FC during (**K**) brown and (**L**) white adipogenesis (D5/D0). The red line represents the genes related to mitochondria GO terms while the black line represents total detectable genes as the background. (**M**,**N**) The cumulative fraction curves of (left) RNA-, (middle) RPF- and (right) Efficiency FCs during adipogenesis for (**M**) all detectable genes and (**N**) genes related to mitochondrion GO. (**O**,**P**) As described in K and L. The red line represents the lipid metabolism related genes. *Represents P value < 0.05; **represents P value < 0.01; ***represents P value < 0.001 (Kolmogorov–Smirnov test). (**Q**) Genes are ranked based on their difference between of Eff-FC in brown and in white adipogenesis. GSEA analysis was performed to derive the P values and normalized enrichment score (NES) for each pathway. Inverse volcano plot was generated using the -log_2_(P-value) vs. NES. (**R**) mRNA expression and (**S**) protein level of Acad9 and Ndufaf1 examined by real-time PCR and Western blot during brown and white adipogenesis. The Western image was cropped from a full gel picture (Fig. S6) N = 3.

We aimed to depict a picture of gene expression changes during adipogenesis at translational and transcriptional levels. First, we examined the distribution of RPF and mRNA fold-changes (FCs) in both brown and white adipogenesis. During brown adipogenesis, the FCs of RPF were more broadly distributed than those of mRNAs (Fig. 2A), while during white adipogenesis (Fig. 2B), the FCs of RPFs and mRNAs were distributed similarly, suggesting that translational control is more involved during brown adipogenesis.

To assess the relative contribution of translation and mRNA to total protein synthesis, we calculated the ribosome loading density which reflects the translational efficiency (referred to as Eff) for each gene as the ratio of RPF to RNA. The correlation between fold changes (FC) in RPF and RNAs manifested a R^2^ of 0.67 for BAT and 0.70 for WAT (Fig. 2C,D), demonstrating a predominant contribution of RNA to total translational change during adipogenesis. Interestingly, we detected a moderate correlation between FCs of RPF and FCs of Eff during BAT adipogenesis (R^2^ = 0.22) but not during WAT adipogenesis (Fig. 2E,F), implying that translational control may contribute more to brown adipogenesis than to white adipogenesis.

959 and 513 genes showed more than 4-FC during brown and white adipogenesis, respectively. We classified them into 12 subgroups according to their relative contribution of Eff and RNA to RPF FCs. Namely, group 1–3 (389 genes) and group 7–9 (237 genes) consist of genes whose RPFs were up-regulated during brown and white adipogenesis, respectively; groups 4–6 (570 genes) and group 10–12 (276 genes) were down-regulated during brown and white adipogenesis, respectively (Fig. 2G and H). Genes in groups 1, 4, 7, 10 were mainly driven by Eff FC; group 3, 6, 9, 12 were mainly regulated by RNA FC; group 2, 5, 8, 11 were synergistically driven by both RNA FC and Eff FC. In summary, 111 (11.6%) and 257 (26.8%) genes were transnationally driven and synergistically driven during brown adipogenesis, respectively (Fig. 2I), while only 14 (2.7%) and 68 (13.3%) genes were translationally driven and synergistically driven during white adipogenesis, respectively (Fig. 2J). In addition, we quantified the relative contribution from Eff and RNA to RPF FCs as previously described^31^ using the ratio of correlations between variables to the correlation between experimental duplicates (Figure S3D), and observed ~30% and ~22% translational contribution during brown and white adipogenesis, respectively. Although the interpretation of these analysis may be limited by the small number of replicates, translational control seems to have a greater influence on total translational changes in brown than white adipocytes.

### The translational efficiency of mitochondria-related genes increases during brown adipogenesis

Although translational control was not the major contributor to changes in protein expression, we sought to assess whether specific pathways were regulated by translational control by performing Gene Ontology analysis for each gene set (Tables 1, 2). For the RNA-driven groups (3, 6, 9,12), we observed similar GOs during brown and white adipogenesis, including oxidation reduction, mitochondrion as up-regulated GOs, and cell adhesion, extracellular region as down-regulated GOs. But genes in translational driven groups were enriched for different GOs in two lineages. For example, chromatin related GOs were down-regulated during brown adipogenesis while translation and ribosome related GOs were down-regulated during white adipogenesis (Tables 1, 2). An interesting observation was that genes in mitochonda-related GOs were significantly over-represented in the synergistic group during brown (Group 2) but not white adipogenesis (Group 8) (Tables 1, 2), suggesting that enhanced translation contributes to the induction of mitochondria genes in brown adipogenesis.

**Table 1:** classification of genes with regulated total translation during brown adipogenesis and their associated GOs.

Gene sets	1	2	3	4	5	6
Category	Translation driven	Synergistic	RNA driven	Translation driven	Synergistic	RNA driven
Number	4	54	331	107	203	260
GO_BP	N.A	Cofactor metabolic process; lipid biosynthetic process; protein folding	Oxidation reduction; electron transport chain; cellular respiration	Chromatin assembly; protein-DNA complex assembly	Translation; polysaccharide biosynthetic process	Cell adhesion; extracellular structure organization
GO_CC	N.A	Mitochondrion	Mitochondrial membrane part; proton-transporting ATP synthase complex	Nucleosome; protein-DNA complex	Ribosome; small ribosomal subunit	Extracellular region

**Table 2:** classification of genes with regulated total translation during white adipogenesis and their associated GOs.

Gene sets	7	8	9	10	11	12
Category	Translation driven	Synergistic	RNA driven	Translation driven	Synergistic	RNA driven
Number	0	7	230	14	61	201
GO_BP	N.A	N.A	Oxidation reduction; generation of precursor metabolites and energy; fatty acid metabolic process	Translation	Cytoskeleton organization; regulation of cell development	Cell adhesion; actin cytoskeleton organization
GO_CC	N.A	N.A	Mitochondrion	Ribosome; ribonucleoprotein complex	Ribosome	Extracellular region

To evaluate the translational regulation of mitochondria genes in a more systemic manner, we plotted the cumulative density functions of FCs (D5/D0) for all mitochondrion GO-related genes (183 genes), with a comparison to all detectable genes as a control. The mitochondrion curve shifted to the right compared to the total gene curve in all RNA, RFP and Eff plots during brown adipogenesis (Fig. 2K), indicating that both mRNA and Eff of mitochondria genes, compared with other genes, were increased during brown adipogenesis. In contrast, although the RPFs and RNAs of mitochondrion related genes were enhanced during white adipogenesis, the translational Effs were relatively repressed (Fig. 2L). Furthermore, we directly compared brown and white adipogenesis in term of FC distribution of mitochondrion related genes as well as total genes. Total genes did not show significant difference at RNA, RFP or Eff (Fig. 2M), but mitochondria genes exhibited significant higher Eff and RFP during brown adipogenesis (Fig. 2N). Taken together, these data demonstrate that both mRNA and Eff synergistically contribute to the induction of mitochondria genes during brown adipogenesis, but not white adipogenesis.

A feature common in both brown and white adipogenesis is the increase of lipid content. We also examined the cumulative fraction curves of FCs for 157 genes in lipid metabolism GOs. Although these genes outperform background genes regarding their induction at RNA and RPF levels (Fig. 2O,P), their Effs are not different from those of total genes in both BAT and WAT adipogenesis, indicating that lipid metabolism genes are mainly governed by RNA changes.

The analysis above has demonstrated that translation control played a crucial role in mitochondria genes during brown adipogenesis. To assess wheather other pathways were regulated at translational levels, we compared the translational changes of brown and white adipogenesis by ranking genes according to their differential FCs (∆FC_Eff_ = (Eff_BATD5_/Eff_BATD0_)/(Eff_WATD5_/Eff_WATD0_)) during brown and white adipogenesis. We performed GSEA on this ranked gene list and plotted the enrichment score (NES) *vs*. P-value for each GO (Fig. 2Q, Figure S3E–G). Consistently with earlier observation, all mitochondria-related GOs ranked as the most up-regulated pathways, indicating that the main targets of translational regulation during brown adipogenesis were the genes involved in mitochondria function. The extracellular matrix related GOs were within the most down-regulated terms, indicating the involvement of translational control in the different cellular matrix between these two systems.

Due to the small number of replicates (duplicates) in this study, our conclusions based on the high throughput analysis may be biased by some sample. To provide experimental evidence supporting our conclusion, we chose two mitochondria genes Acad9 (Acyl-CoA Dehydrogenase Family Member 9) and Ndufa1 (NADH:Ubiquinone Oxidoreductase Subunit A1), and performed Real-time PCR and Western to examine their mRNA and protein changes during brown and white adipogenesis. Based on ribosome profiling and and RNA-seq data, their mRNA FCs should increase similarly during both brown and white adipogenesis, but their RFP FCs were induced more significantly during brown adipogenesis (Supplemental File1). The real-time PCR analysis showed that Acad9 mRNA was increased by ~6 fold during brown and white adipogenesis, and its expression level was similar in mature brown and white adipocytes (D5)(Fig. 2R). In contrast, although the protein level of Acad9 examined by Western exhibited a significant increase during both brown and white adipogenesis, it reached a higher level in brown adipocytes than in white adiopocytes (Fig. 2S), suggesting a higher translational efficiency in brown adipocytes. Consistent with the prediction based on our transcriptome and translatome analysis, our experimental evidence suggests that the conclusions drawn from our high-throughput analysis can reflect the translational difference between brown and white adipogenesis at least for a set of genes involved in mitochondria functions.

### Translational control contributes to the total translational difference between BAT and WAT

Before assessing the contribution of translational control to the molecular divergence between BAT and WAT, we tested whether the RPF FCs can faithfully reflect the protein FCs by directly quantifying protein abundance with ITRAC (isobaric tags for relative and absolute quantification). As expected, GESA reveals significant up-regulation of oxidative phosphorylation and mitochondrion GO (Figure S4A,B). In addition, we compared the regulated pathways based on the FCs of RPF, RNA and proteins and found that they shared many pathways related to the distinct feature between BAT and WAT (Figure S4C). To determine the contribution of RPF and RNA to protein difference between BAT and WAT, we examined the correlation between protein FCs and RFP FCs, and protein FCs and RNA FCs (Figure S4D,E). Both RPF FCs and RNA FCs correlated well with protein FCs (R^2^ = 0.5, R^2^ = 0.51).

**Figure 3:**
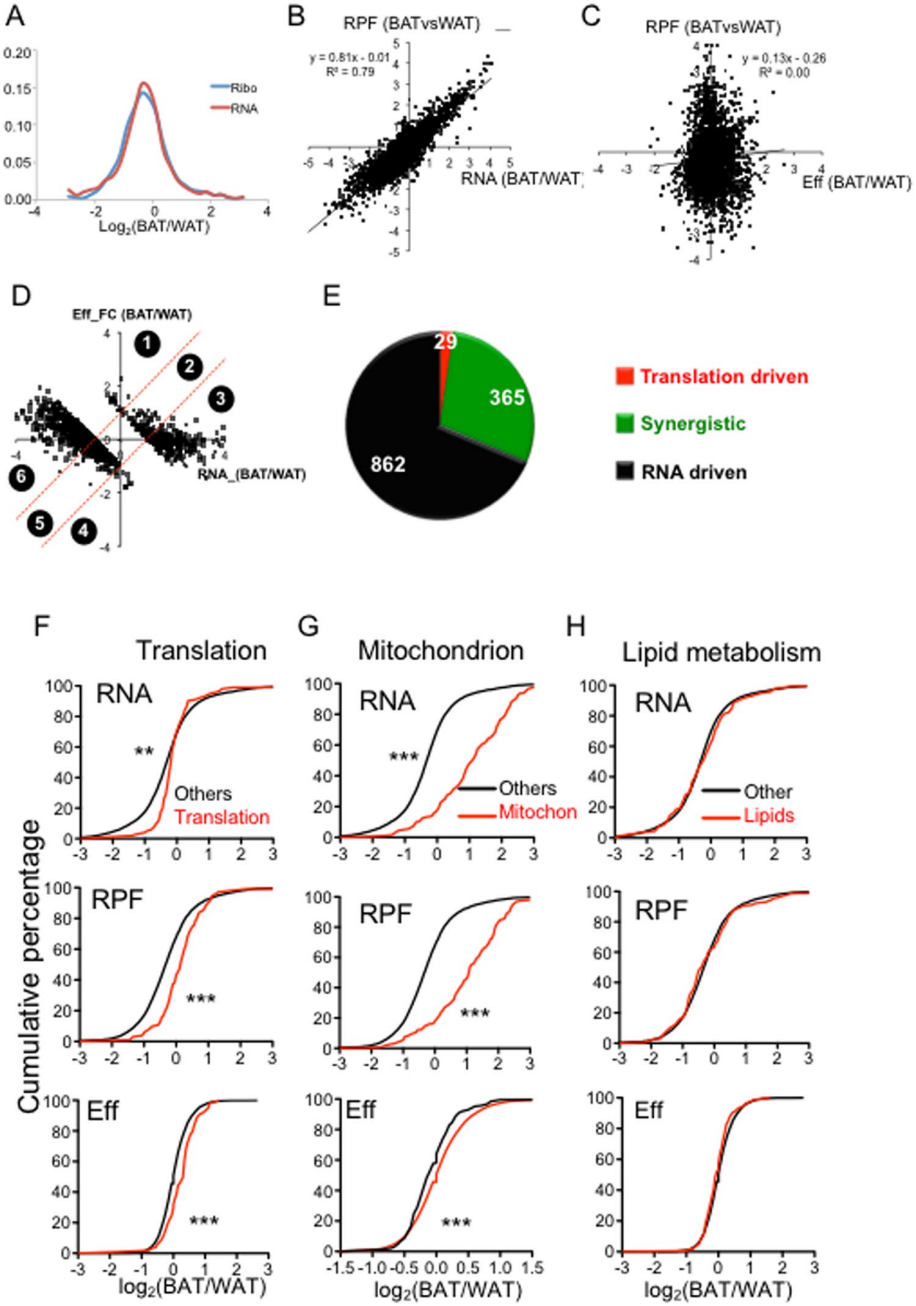
Comparison of the translational profiles between BAT vs WAT. (**A**) The distribution of RPF-FCs and RNA-FCs between BAT vs WAT. (**B**) Correlation between RPF-FC and RNA-FC in BAT vs. WAT. (**C**) Correlation between RPF-FCs and Eff-FCs in BAT vs. WAT. (**D**) Based on the relative contribution of mRNA-FCs vs Eff-FCs to the total translation FCs, genes with ≥2 fold RPF changes between BAT vs WAT were classified into 6 groups. Genes with higher and lower RFP in BAT comprise Group 1–3 and Group 4–6, respectively. (**G**) Pie chart to show the composition of each group. Genes were classified into three types: translation driven, synergistic (driven by both RNA and translation), and RNA driven. (**F**) The cumulative fraction curves of RNA- (upper), RPF- (middle) and Efficiency-(lower) FCs between BAT and WAT. The red and blue lines represent the gene population involved in translation and the total gene population, respectively. (**G**,**H**) Similar to H. The red line represents the genes involved in (**G**) mitochondrion and (**H**) lipid metabolism. *Represents P value < 0.05; **represents P value < 0.01; ***represents P value < 0.001 (Kolmogorov–Smirnov test).

To determine the role of translational control in the lineage difference between BAT and WAT, we first examined the distribution of RNA FCs and RPF FCs between BAT and WAT (Fig. 3A). We observed no significant difference, suggesting that mRNA levels were the major contributor to RPF changes. Moreover, RPF FC and RNA FCs showed a clear positive correlation with a R^2^ of 0.79 (Fig. 3B), while the RPF FC didn’t correlate with Eff FCs (Fig. 3C), suggesting that the change at RNA level is the predominant contributor to the RPF difference between BAT and WAT.

As described in the adipogenesis analyses, we classified genes with RPF FC ≥ 2 according to the relative contribution from Eff and RNA. Although a small fraction of genes (2.3%) were driven by translational change alone, 29.1% genes were synergistically driven by both RNA and Eff changes (BAT/WAT) (Fig. 3E). GO analysis revealed a significant enrichment of translation/ribosome GO in group 1 and mitochondrion GO in group 2, suggesting that genes involved in these terms were more active in translation in BAT (Table 3). To systematically test this observation, we extracted all genes from two GOs as well as lipid metabolism pathways and plotted their cumulative distribution curves in comparison to all other genes. The curves representing genes related to translation and mitochondria GOs shifted to the right side compared with other genes at RNA, RPF and Eff levels, indicating that the genes in these pathways tend to be more actively transcribed and translated (Fig. 3F,G). In contrast, lipid metabolic genes show no significant difference from other genes (Fig. 3H).

**Table 3:** Classification of genes with differential total translation between BAT vs WAT and their associated GOs.

Gene sets	1	2	3	4	5	6
Category	Translation driven	Synergistic	RNA driven	Translation driven	Synergistic	RNA driven
Number	19	88	288	10	277	574
GO_BP	Translation	Transmembrane transport; cell-cell signalling	Electron transport; oxidation reduction	N.A	Cytoskeleton metabolic process	Cell adhesion; inflammatory response
GO_CC	Ribosome	Mitochondrion; ribosome	Mitochondrion	N.A	Golgi apparatus part	Extracellular region

### The total translation of microRNAs’ targets is repressed in both WAT and BAT

**Figure 4:**
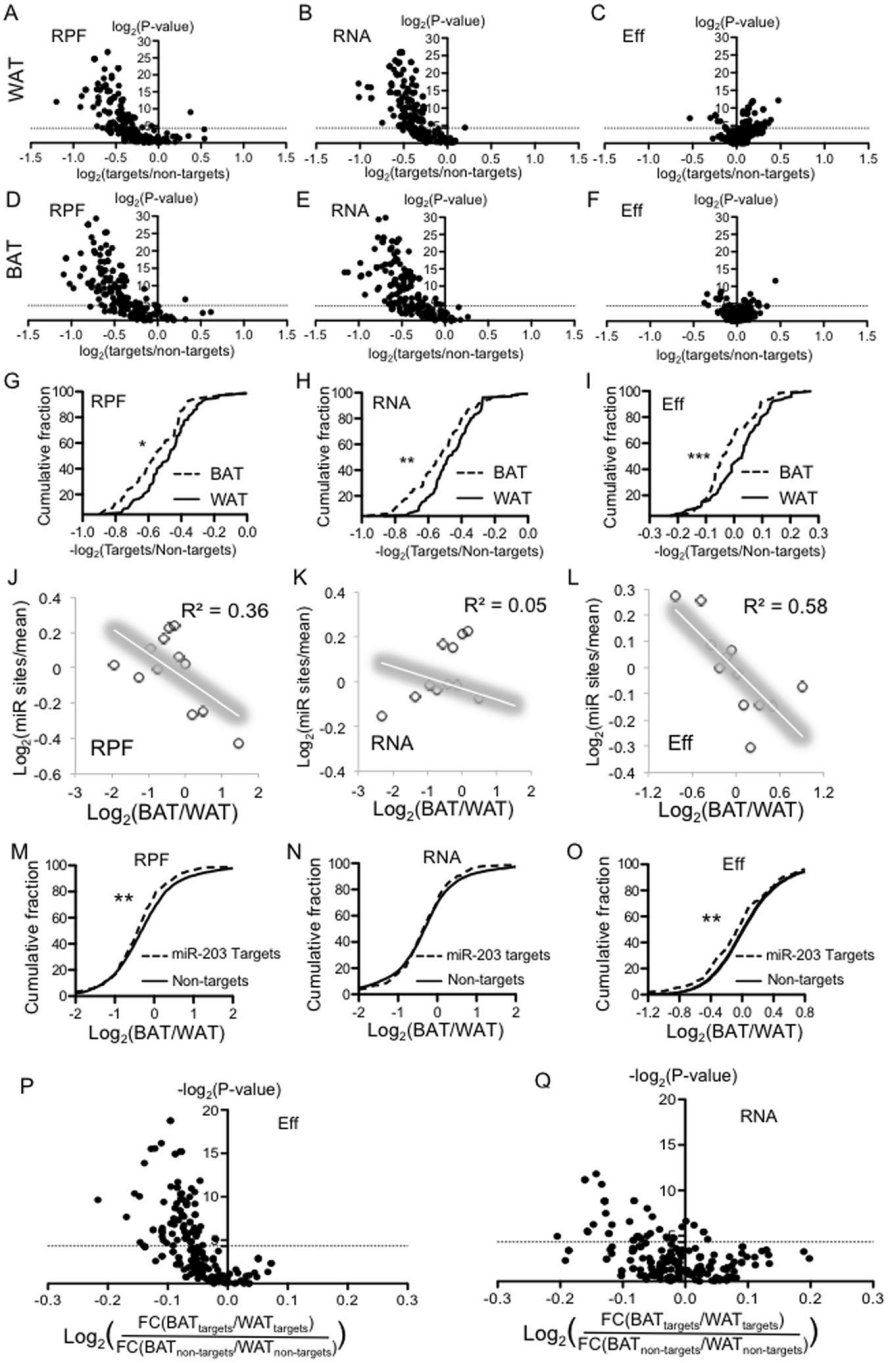
microRNAs contribute to the differential translation between BAT and WAT. (**A**–**C**) For each microRNA, P values for the cumulative distribution of its target vs. non-targets expression were calculated by KS test. An example is illustrated in Fig. 4SA–C. The P values were plotted (y-axis) againt the FCs of targets’ mean value vs. non-targets’ mean value (x-axis). (**A**) Based on RPF; (**B**) Based on RNA expression; (**C**) based on translational efficiency. (**A**–**C**) Were derived from WAT data. (**D**–**F**) Similar to A–C, but were decrived from BAT data. (**G**–**I**) Comparision of the target repression in BAT vs WAT at (**G**) RPF, (**H**) RNA and (**I**) Eff level. For microRNAs whose targets are signficantly repressed in WAT or BAT in term of total translational output (RPF), the ratio of targets’ mean value vs non-targets’ mean value were calculated. The culmulative curves of these ratios (target/non-target) in BAT and WAT were plotted. (**J**–**L**) MicroRNA binding sites distribution in mRNAs binned according to their expression ratios between BAT and WAT. (**J**) Based on RPF (**K**) based on RNA and (**L**) based on Eff. Data points were fit into a linear model. (**M**–**O**) The FCs in BAT vs WAT for each mRNA was calcuated. (**M**) Was based on RFP, (**N**) based on RNA, (**O**) based on Eff. The culmulative curves for miR-203 targets’ FCs and non-targets’ FCs were plotted. KS test was used to calculated P value. (**P**,**Q**) As decribed in M, the KS test P values for the differential distribution between targets vs. non-targets were calculated for each microRNA. The P values were plotted as y-axis. For each microRNA, the FCs of targets (or non-targets) between BAT and WAT were calculated. The ratios between FC of targets and FC of non-targets were plotted as the value in x-axis. (**P**) is based on Eff and (**Q**) is based on RNA. *Represents P value < 0.05; **represents P value < 0.01; ***represents P value < 0.001 (Kolmogorov–Smirnov test).

microRNAs are endogenous small non-coding RNAs that can bind to its mRNA targets and result in RNA decay or translational repression^32–34^. We suspected that the regulatory interaction microRNAs and its mRNA targets might partially account for the differential gene expression in BAT and WAT. To address this possibility, we plotted the cumulative fraction curves for the expression of each microRNA’s conserved targets and non-targets to calculate the P value for their distribution within each tissue. Conserved microRNAs target lists for each microRNA were derived from TargetScan^35^. Since microRNAs in the same family harbor the same seed sequences and therefore target the same set of mRNAs, we considered different members in a microRNA family as one microRNA to reduce the redundancy. 219 conserved microRNAs were included in this assay. For example, the targets of miR-203 significantly shift to the downwards in RPF and RNA plots (Figure S5A,B,), indicating that its targets, in comparison with non-targets, were repressed in BAT. We observed no significant change at the Eff levels (Figure S5C). Thus, the repression of miR-203 targets was mainly due to reduced RNA levels. For each microRNA, we plotted the P value from targets vs. non-targets distribution, and the ratio of average expression between targets vs. non-targets. In both BAT and WAT, the majority of microRNAs exert a repressive influence on their targets at the RNA and RPF levels (Fig. 4A,B,D,E), but not at the translational level (Eff) (Fig. 4C,F).

### Most miRNAs repress targets more in BAT than in WAT

To compare the repressive effect of each microRNA in BAT and WAT, we plotted their cumulative curves of the ratio between targets vs non-targets at RPF, RNA and Eff levels (Fig. 4G,H,I). The BAT curves were appreciably shifted towards the left by all three metrics. Thus, the repressive extent of microRNAs is stronger in BAT than WAT. Quantitatively, 70.5% and 77.0% microRNAs show stronger repression in BAT at RNA and Eff, respectively (Figure S4D)

From another perspective, we examined the microRNA binding sites distribution across mRNAs that were ranked by their FCs between BAT and WAT. Interestingly, genes with lower RPF FCs and Eff FCs tend to harbors more microRNA binding sites (Fig. 4J,L), but this trend was not observed with RNA FCs (Fig. 4K), supporting that genes with lower translational efficiency in BAT might be due to their interactions with more microRNAs.

We have analyzed the regulatory interactions of each microRNA in BAT and WAT separately, and have employed the ratio between targets and non-targets to evaluate the extent of target repression within each tissue (Fig. 4G,H,I). Next, we directly compared microRNA targets’ expression in BAT and in WAT by plotting the cumulative curves of the FCs between BAT and WAT at RPF, RNA and Eff levels. For example, the curves representing miR-203’s targets shifted to the left relative to non-target curve at RPF (Fig. 4M) and Eff (Fig. 4O) levels but not RNA level (Fig. 4N), indicating that the translational Effs of miR-203’s target genes were lower in BAT than in WAT.

To examine the repressive effect of each microRNA in different depots, we calculated the FCs of targets (or non-targets) between BAT and WAT as well as the P value from targets vs. non-targets distribution comparison for each microRNA. The difference in the FC of targets (BAT/WAT) and the FC of non-targets (BAT/WAT), was plotted against P value of each microRNA (Fig. 4P,Q). For most of microRNAs with significant P-values, their target genes were more repressed than non-target genes in BAT at the translational level (Fig. 4P) and RNA levels (Fig. 4Q). Taken together, our studies demonstrate that microRNA targets were repressed to a greater extent in BAT than in WAT which suggests that microRNAs are more active in BAT. Consistent with this conclusion, others and our group have genetically deleted key components in microRNA biogenesis such as Dicer^25^ or Dgcr8^26^ in adipose and the microRNA-depleted BAT manifests WAT-like phenotypes including an enlarged organ mass, a pale colour, bigger lipid-droplets, and decreased expression of BAT markers.

In present study, we have provided an integrative view of translatome by ribosome-profiling and transcriptome by RNA-seq during lineage-specific adipogenesis and between different depots. Our comparative analyses reveal a preferentially enhanced translation efficiency of mitochondria genes during brown adipogenesis and in BAT compared with WAT. Through systemic analysis of microRNAs’ regulatory interactions, we conclude that the targets of most microRNAs tend to have lower translational efficiency in BAT and microRNAs’ regulatory interaction can make important contribution to the differential translational profile between BAT and WAT. However, some of these conclusions may be biased by the small number of our replicates (duplicates) and the full validity of these conclusions will require further investigation.

## Methods

### Animal

C57BL6 mice were purchased from InVivos and subsequently hosted at the animal vivarium at DUKE-NUS Medical School. All animal experimental protocols were approved by the Singapore SingHealth Research Facilities Institutional Animal Care and Use Committee (2016/SHS/1179). All methods were performed in accordance with the relevant guidelines and regulations.

### RNA-seq, ribosome profiling and Itraq for adipose tissue

6 male mice were sacrificed at 6-weeks old to harvest their interscapular brown adipose tissue (BAT) and epididymal white adipose tissue (WAT). Tissues from 3 mice were pooled together as one replicate. Tissues were minced into small pieces and separated into 3 shares for RNA-seq, iTRAQ mass spec and ribosome profiling.

For RNA-seq, total RNAs were extracted with miRNeasy Mini Kit (Qiagen) and RNA libraries were prepared with NEBNext® Ultra™ RNA Library Prep Kit for Illumina (E7530S). RNA-seq was performed at the Illumina HiSeq2000 platform in BGI.

For iTRAQ mass spec, brown or white adipose tissue was homogenized by MagNA lyser (Roche) in 1.5 ml RIPA buffer RIPA buffer (50 mM Tris-HCl, pH 7.4, 1% NP-40, 0.5% Na-deoxycholate, 0.1% SDS, 150 mM NaCl, 2 mM EDTA, 50 mM NaF) supplemented with Proteinase inhibitors. Tissue lysates were incubated on ice for 10 minutes and centrifuged in a benchtop centrifuge at 12000 rpm for 10 minutes at 4 °C. The fat layer on top was removed and the clear supernatant was collected without disturbing the pellet on the bottom. iTRAQ was performed in Beijing Genomics Institute (BGI).

For ribosome profiling, brown or white adipose tissue were homogenized with pestle and mortar in the presence of liquid nitrogen. The homogenized powder was transferred into 1.5 ml tubes and lysed with 1 ml lysis buffer (1% NP-40, 200 mM KOAc, 25 mM K-HEPES pH 7.2, 14 mM MgCl, and 4 mM CaCl_2_). Samples were incubated on ice for 5 minutes and centrifuged at 9,000 g in a benchtop centrifuge at 4 °C for 10 minutes. The fat layer on top was removed and the 300 ul supernatant was collected. Following steps were conducted according to a published protocol^36^.

### Cell culture

Primary brown and white adipocyte cultures were conducted as described in earlier studies^13, 21, 28^. Briefly, for each batch of cell culture, 6–8 C57BL/6 mice (2–3 weeks old) were sacrificed to harvest interscapular BAT and inguinal WAT. Tissues were minced with scissors and digested in 0.2% collagenase (Sigma). Stromal vascular fraction cells were collected by centrifugation, red blood cells were lysed with NH_4_Cl (1 M) and then the SVF was filtered through a 40 um membrane. SVF cells isolated from both BAT and WAT were cultured to confluence in 4 × 35 mm dishes in DMEM with 10% new-born calf serum and induced to differentiate for 2 days with DMEM containing 10% fetal bovine serum, 850 nM insulin (Sigma), 0.5 uM dexamethasone (Sigma), 250 uM 3-isobutyl-1-methylxanthine, phosphodiesterase inhibitor (IBMX, Sigma), 1 uM Rosiglitazone (Cayman Chemical). The induction medium was replaced with DMEMcontaining 10% FBS and 160 nM insulin for 2 days. Then cells were incubated in DMEM with 10% FBS for another day.

### RNA-seq, ribosome profiling for cell cultures

Brown and white adipocytes were cultured to Day 5 as described above. Cell at day 0 (preadipocytes) and mature adipocytes at day 5 were washed with PBS and harvested for RNA-seq and ribosome profiling as described in earlier study^36^.

### Western Blot

For western blot analyses, cells were lysed in radioimmunoprecipitation assay (RIPA) buffer (0.5% NP-40, 0.1% SDS, 150 mM NaCl, 50 mM Tris-Cl at pH 7.5). Proteins were separated by SDS-polyacrylamide gel electrophoresis, transferred to a nitrocellulose membrane (Millipore) and probed with anti-Ndufaf1, anti-Acad9 antibodies (Proteintech) and beta-tubulin (Cell Signaling).

### Data analysis

A consensus reference transcriptome for all datasets was first generated by using all RNA-seq experiments as input into Tophat and Cufflinks^37^, then selecting the most abundant isoform. Ribosome profiling and RNA-seq reads were mapped to the reference transcriptome using Bowtie and allowing one mapped site per read and no mismatches^38^. The translation or mRNA level of a gene was defined as the density of reads, in reads per kilobase of coding sequence per million mapped reads. Translational efficiency was defined as the RPKM for ribosome footprinting divided by the RPKM for RNA-seq. The single-site position of a read was defined at 14 nt beyond the 5′ end of the read.

### Statistics

For gene ontology analysis, categories were generated as described in Results and statistical significance determined using the hypergeometric test (p-value cutoff of 0.05). Kolmogorov–Smirnov test was used to determine the statistic difference between the cumulative curves of two gene populations. All experiments were performed in accordance with relevant guidelines and regulations.

### Data Availability

Data ﻿can be accessed at GSE89108.

## Electronic supplementary material

Supplementary Information

dataset 1

dataset 2

dataset 3

dataset 4

dataset 5

dataset 6

dataset 7

dataset 8

dataset 9

dataset 10

dataset 11

dataset 12
