# Supplementary material for: Integrative analyses of translatome and transcriptome reveal important translational controls in brown and white adipose regulated by microRNAs
Source: Sci Rep. 2017 Jul 18;7:5681. doi: 10.1038/s41598-017-06077-3 (PMC5515893; doi:10.1038/s41598-017-06077-3)
Supplement: Supplementary file 1 — Supplementary Information [file 41598_2017_6077_MOESM1_ESM.pdf]

Integrative analyses of translatome and transcriptome reveal important translational controls in brown and white adipose regulated by microRNAs

David W. Reid[1,6], Dan Xu[1,4,6*], Peng Chen[5], Hongyuan Yang[3], Lei Sun[1,2*]

[1]Cardiovascular and Metabolic Disorders Program, Duke-NUS Graduate Medical School, 8 College Road, Singapore 169857, Singapore.
[2]Institute of Molecular and Cell Biology, 61 Biopolis Drive, Proteos, Singapore 138673, Singapore
[3]School of Biotechnology and Biomolecular Sciences, University of New South Wales, Sydney 2052, Australia.
[4]School of Laboratory Medicine and Life Science, Wenzhou Medical University, Wenzhou, Zhejiang 325035, China
[5]Division of Bioengineering, Nanyang Technological University, 70 Nanyang Drive, Singapore 637457, Singapore
[6]These authors equally contributed to this work.
* Correspondence: sun.lei@duke-nus.edu.sg (L.S), danxuwzmc@qq.com (D.X)

Supporting Information Legends

Figure S1. Characterization of the adipocyte differentiation system.

(A) ORO staining picture of the reprehensive samples during brown and white adipogenesis time course. (B) Key adipogenic marker (Fabp4 and Pparg) expression during the differentiation time course detected by realtime PCR. N=4. Primary adipocyte precursors were isolated as described in methods. ORO staining and realtime PCR were performed at the indicated time point after induction of differentiation.

Figure S2. Correlation of ribosome profiling and/or RNA-seq between different samples

(A) Correlation of RNA-seq data between replicates.

(B) Correlation of ribosome profiling data between replicates.

(C-E) GSEA analysis of Oxidative phosphorylation across the ranked genes based on their RNA FCs during brown adipogenesis (C), white adipogenesis (D) and BAT vs. WAT (E).

(F) Heatmap of the correlation efficiency for ribosome profiling data between different samples.

(G) Heatmap of the correlation efficiency for RNA-seq data between different samples.

Figure S3. Translational control contributes to gene expression change during adipogenesis.

(A) Heatmap of genes with ≥4 RPF FCs during both brown and white adipogenesis.

(B) Correlation of RPF FCs during white adipogenesis and brown adipogenesis.

(C) Correlation of RNA FCs during white adipogenesis and brown adipogenesis.

(D) The relative contribution of RNA change and Eff change to the total translational change.

(E,F,G) GSEA of ranked genes based on $\log2\Delta FC_{Eff}=(Eff_{BATD5}/Eff_{BATD0})/(Eff_{WATD5}/Eff_{WATD0})$ in brown and in white adipogenesis.

Figure S4. Comparison of proteomics between BAT vs WAT by ITRAQ.

(A,B) GSEA of genes ranked by their protein FCs between BAT and WAT.

(C) GSEA were performed using ranked genes based on their RPF-, RNA-, and protein FCs between BAT and WAT. Normalized enrichment scores (NES) of GSEA pathways significantly altered in all three analysis (FDR<0.25) are plotted in a heatmap.

(E) Correlation between protein FCs and RPF FCs in BAT vs. WAT.

(F) Correlation between protein FCs and RNA FCs in BAT vs. WAT.

Figure S5.

(A-C) Cumulative curves of miR-203's targets and non-targets were plotted based on their expression in BAT. (A) based on RPF, (B) base on RNA, (C) based on Eff. The KS test was used for P value.

(D) The FCs of targets vs non-targets were calculated in BAT and in WAT. The stack bar indicates the percentage of miRNAs whose targets are more repressed in BAT (black) and miRNAs whose targets are more repressed in WAT (grey).

The repressive extent of miRNA targets is not related to microRNA abundance.

(B, C) The distribution of (B) RPF and (C) RNA FCs between targets vs non-targets across miRNA abundance derived from small RNA-seq in BAT.

Figure S6. The whole gel of Ndufaf1 and Acad9.

Supplemental datasets

Download from

https://www.dropbox.com/sh/xp3x9ueklzzawqj/AACJibtDRYyOHpKNriVmUjQka?dl=0

S01 Ribo+RNA+eff_Protein_processed 2

Expression values in Ribo, RNA and Protein of each gene in all samples. It also includes the differential expression between different samples.

S02 Gene groups_BATD5vsD0_FC_Ribo>4

Genes with total translation FC >4 during brown adipogenesis are divided into 4 groups as shown in Figure 2G.

S03 Gene groups_WATD5vsD0_FC_Ribo>4

Genes with total translation FC >4 during white adipogenesis are divided into 4 groups as shown in Figure 2H.

S04 Gene groups_BATvsWAT_FC_Ribo>2

Genes with total translation FC >2 between BAT and WAT are divided into 4 groups as shown in Figure 3D.

S05 FC during adipogenesis_mitochondria, Lipids, translation genes

Fold changes (at Ribo, RNA and Eff levels) for Mitochondria, Lipids, Translation GOs-related genes during brown and white adipogenesis, which were used to generate figure 2K-P.

S06 FC between BAT vs WAT_mitochondria, Lipids, translation genes

Fold changes (at Ribo, RNA and Eff levels) for Mitochondria, Lipids, Translation GOs-related genes between BAT and WAT, which were used to generate Figure 3 F-H

S07 Protein_ITRAQ

Protein quantification data in ITRAQ analysis

S08 Number of miRsites vs RPF (BATvsWAT)_binned

The distribution of the conserved miRNA binding sites on mRNAs which were binned into 12 groups according to the Ribo fold changes (FC) between BAT and WAT. Data were used for Figure 4J.

S09 Number of miRsites vs RNA (BATvsWAT)_binned

The distribution of the conserved miRNA binding sites on mRNAs which were binned into 12 groups according to the RNA fold changes (FC) between BAT and WAT. Data were used for Figure 4K.

S10 Number of miRsites vs Eff (BATvsWAT)_binned

The distribution of the conserved miRNA binding sites on mRNAs which were binned into 12 groups according to the Eff fold changes (FC) between BAT and WAT. Data were used for Figure 4L.

S11 FC_P_miR-targets vs non-targets in BAT or WAT

For each microRNA, its target gene expression at RPF, RNA and Eff levels are compared with its non-target gene expression in BAT or WAT.

S12 Targets and non-targets_BATvsWAT

For each microRNA, its target gene expression ratio between BAT and WAT are calculated for non-target genes vs. target genes.

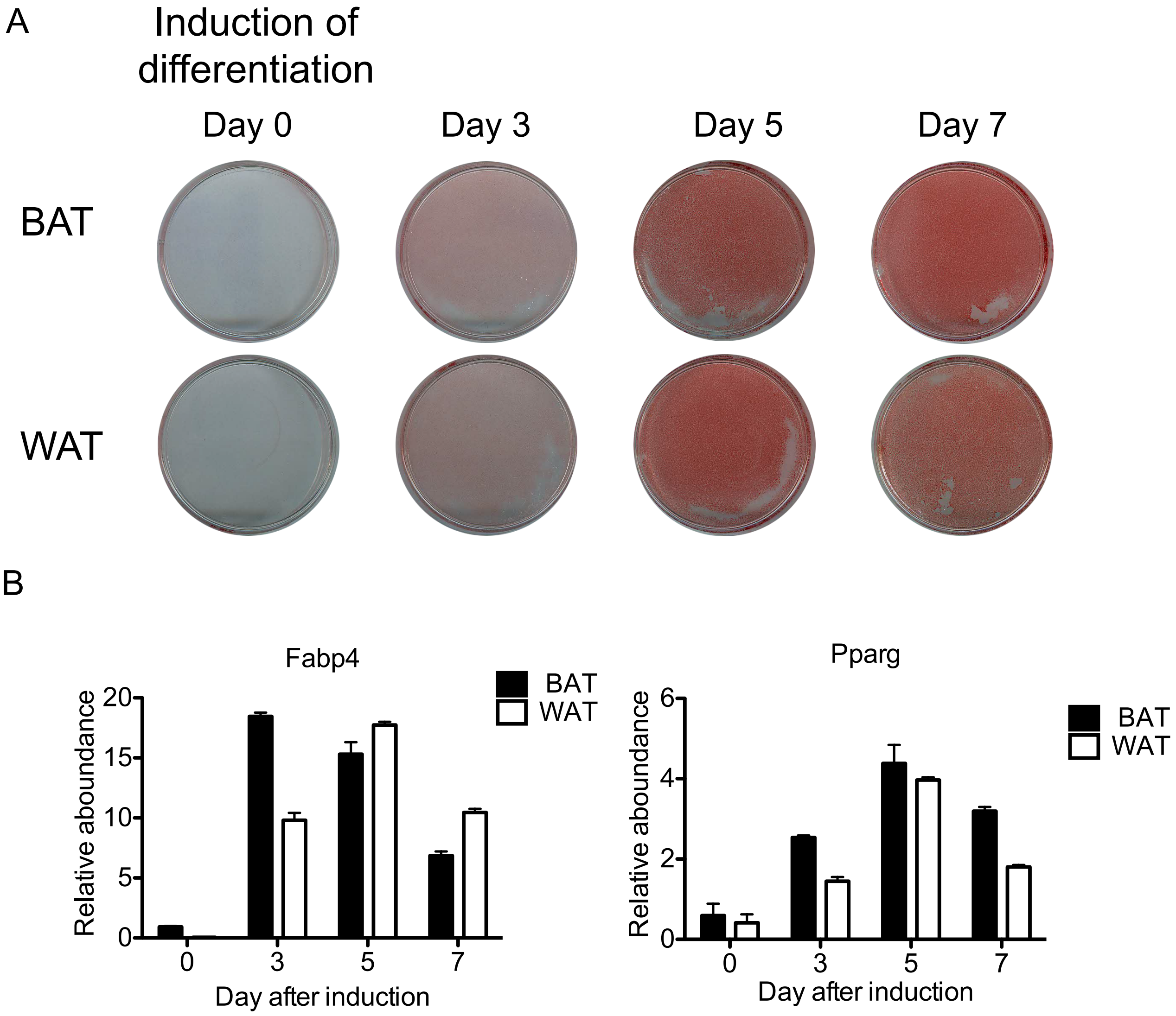
A
Induction of differentiation
Day 0
Day 3
Day 5
Day 7
BAT
WAT
B
Fabp4
Relative aboundance
20
15
10
5
0
0
3
5
7
Day after induction
BAT
WAT
Pparg
Relative aboundance
6
4
2
0
0
3
5
7
Day after induction
BAT
WAT

Figure S1

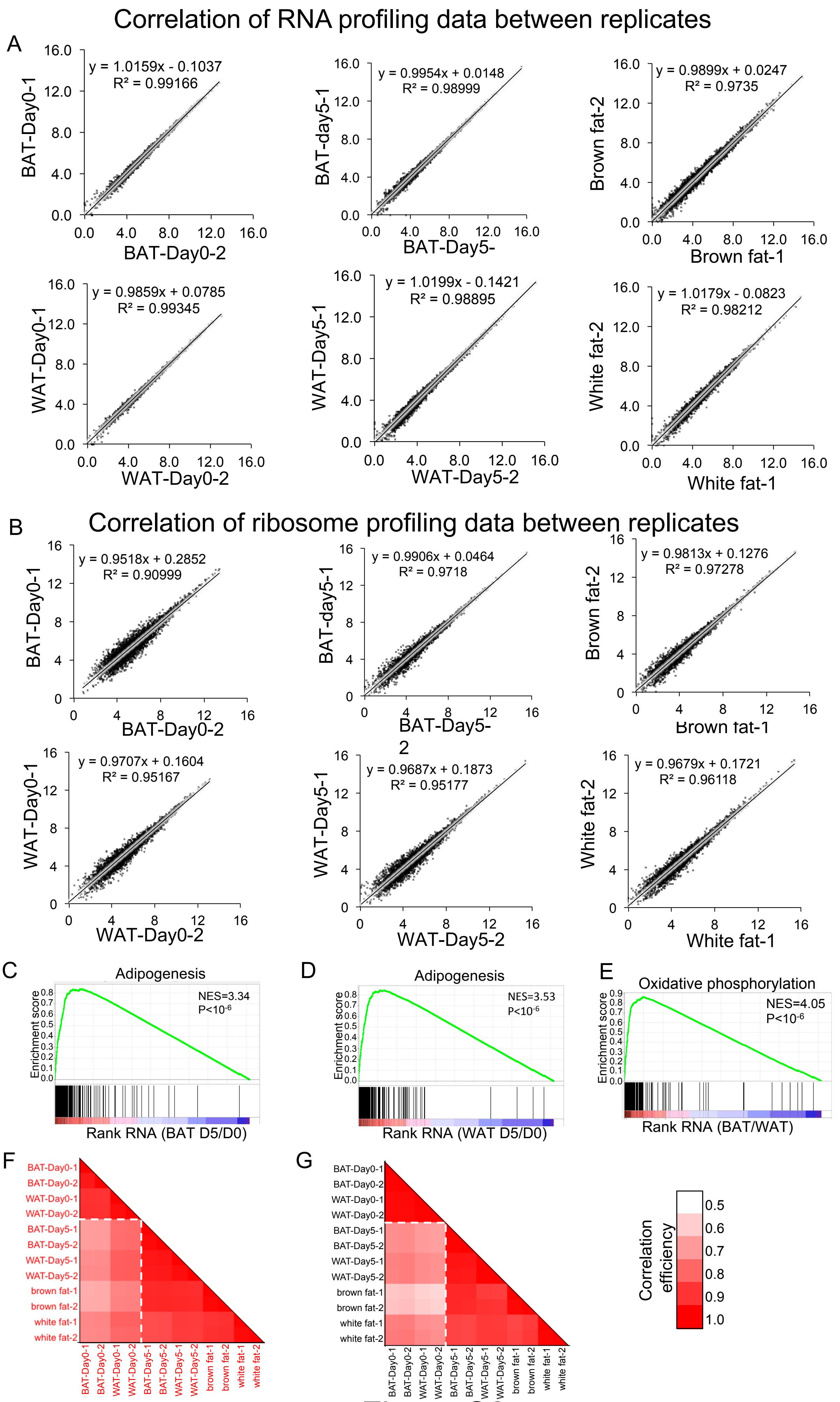
Correlation of RNA profiling data between replicates
A
y = 1.0159x - 0.1037
R² = 0.99166
BAT-Day0-1
BAT-Day0-2
y = 0.9954x + 0.0148
R² = 0.98999
BAT-day5-1
BAT-Day5-
y = 0.9899x + 0.0247
R² = 0.9735
Brown fat-2
Brown fat-1
y = 0.9859x + 0.0785
R² = 0.99345
WAT-Day0-1
WAT-Day0-2
y = 1.0199x - 0.1421
R² = 0.98895
WAT-Day5-1
WAT-Day5-2
y = 1.0179x - 0.0823
R² = 0.98212
White fat-2
White fat-1
Correlation of ribosome profiling data between replicates
B
y = 0.9518x + 0.2852
R² = 0.90999
BAT-Day0-1
BAT-Day0-2
y = 0.9906x + 0.0464
R² = 0.9718
BAT-day5-1
BAT-Day5-2
y = 0.9813x + 0.1276
R² = 0.97278
Brown fat-2
Brown fat-1
y = 0.9707x + 0.1604
R² = 0.95167
WAT-Day0-1
WAT-Day0-2
y = 0.9687x + 0.1873
R² = 0.95177
WAT-Day5-1
WAT-Day5-2
y = 0.9679x + 0.1721
R² = 0.96118
White fat-2
White fat-1
C
Adipogenesis
NES=3.34
P<10-6
Enrichment score
Rank RNA (BAT D5/D0)
D
Adipogenesis
NES=3.53
P<10-6
Rank RNA (WAT D5/D0)
E
Oxidative phosphorylation
NES=4.05
P<10-6
Rank RNA (BAT/WAT)
F
G
BAT-Day0-1
BAT-Day0-2
WAT-Day0-1
WAT-Day0-2
BAT-Day5-1
BAT-Day5-2
WAT-Day5-1
WAT-Day5-2
brown fat-1
brown fat-2
white fat-1
white fat-2
Correlation efficiency
0.5
0.6
0.7
0.8
0.9
1.0

Figure S2

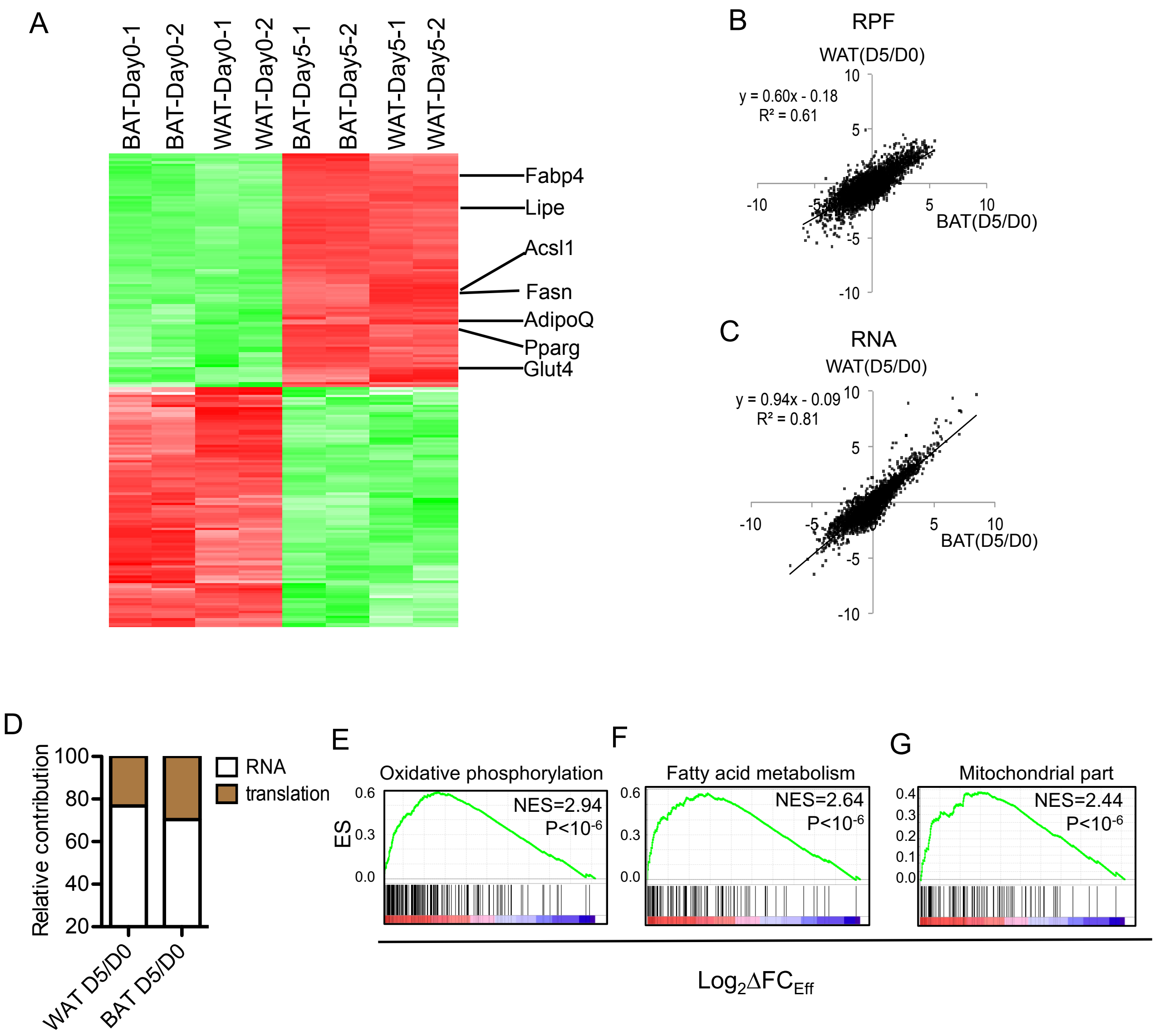
A
BAT-Day0-1
BAT-Day0-2
WAT-Day0-1
WAT-Day0-2
BAT-Day5-1
BAT-Day5-2
WAT-Day5-1
WAT-Day5-2
Fabp4
Lipe
Acsl1
Fasn
AdipoQ
Pparg
Glut4
B
RPF
WAT(D5/D0)
y = 0.60x - 0.18
R² = 0.61
BAT(D5/D0)
C
RNA
WAT(D5/D0)
y = 0.94x - 0.09
R² = 0.81
BAT(D5/D0)
D
Relative contribution
RNA
translation
WAT D5/D0
BAT D5/D0
E
Oxidative phosphorylation
NES=2.94
P<10-6
ES
F
Fatty acid metabolism
NES=2.64
P<10-6
G
Mitochondrial part
NES=2.44
P<10-6
Log2ΔFCEff

Figure S3

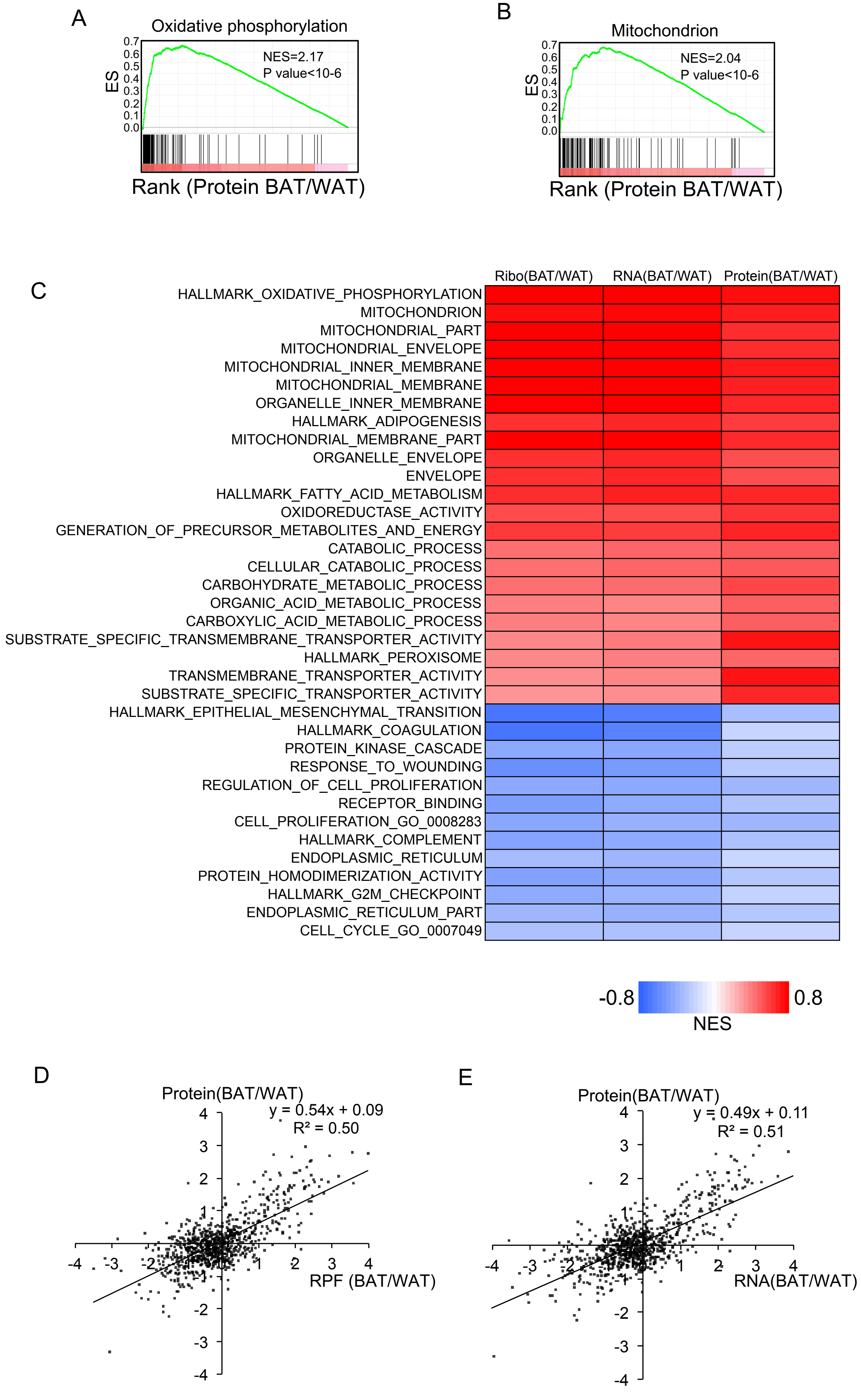
A
Oxidative phosphorylation
ES
NES=2.17
P value<10-6
Rank (Protein BAT/WAT)
B
Mitochondrion
ES
NES=2.04
P value<10-6
Rank (Protein BAT/WAT)
C
Ribo(BAT/WAT)
RNA(BAT/WAT)
Protein(BAT/WAT)
HALLMARK_OXIDATIVE_PHOSPHORYLATION
MITOCHONDRION
MITOCHONDRIAL_PART
MITOCHONDRIAL_ENVELOPE
MITOCHONDRIAL_INNER_MEMBRANE
MITOCHONDRIAL_MEMBRANE
ORGANELLE_INNER_MEMBRANE
HALLMARK_ADIPOGENESIS
MITOCHONDRIAL_MEMBRANE_PART
ORGANELLE_ENVELOPE
ENVELOPE
HALLMARK_FATTY_ACID_METABOLISM
OXIDOREDUCTASE_ACTIVITY
GENERATION_OF_PRECURSOR_METABOLITES_AND_ENERGY
CATABOLIC_PROCESS
CELLULAR_CATABOLIC_PROCESS
CARBOHYDRATE_METABOLIC_PROCESS
ORGANIC_ACID_METABOLIC_PROCESS
CARBOXYLIC_ACID_METABOLIC_PROCESS
SUBSTRATE_SPECIFIC_TRANSMEMBRANE_TRANSPORTER_ACTIVITY
HALLMARK_PEROXISOME
TRANSMEMBRANE_TRANSPORTER_ACTIVITY
SUBSTRATE_SPECIFIC_TRANSPORTER_ACTIVITY
HALLMARK_EPITHELIAL_MESENCHYMAL_TRANSITION
HALLMARK_COAGULATION
PROTEIN_KINASE_CASCADE
RESPONSE_TO_WOUNDING
REGULATION_OF_CELL_PROLIFERATION
RECEPTOR_BINDING
CELL_PROLIFERATION_GO_0008283
HALLMARK_COMPLEMENT
ENDOPLASMIC_RETICULUM
PROTEIN_HOMODIMERIZATION_ACTIVITY
HALLMARK_G2M_CHECKPOINT
ENDOPLASMIC_RETICULUM_PART
CELL_CYCLE_GO_0007049
-0.8
0.8
NES
D
Protein(BAT/WAT)
y = 0.54x + 0.09
R² = 0.50
RPF (BAT/WAT)
E
Protein(BAT/WAT)
y = 0.49x + 0.11
R² = 0.51
RNA(BAT/WAT)

Figure S4

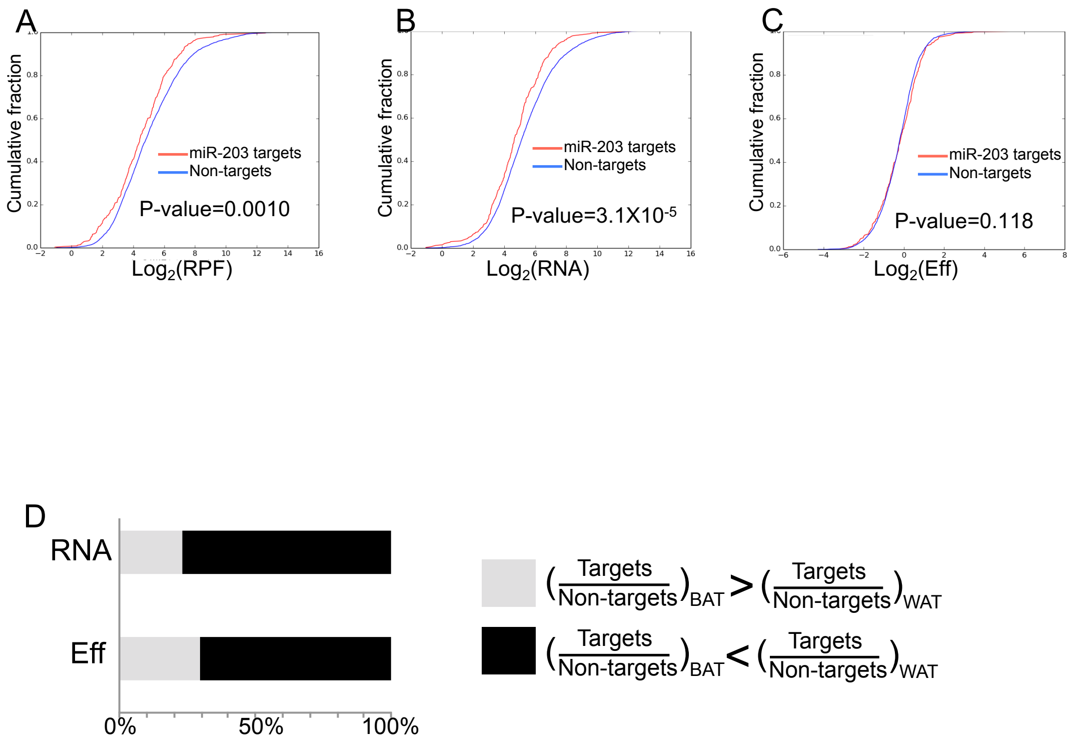
A
Cumulative fraction
miR-203 targets
Non-targets
P-value=0.0010
Log2(RPF)
B
Cumulative fraction
miR-203 targets
Non-targets
P-value=3.1X10-5
Log2(RNA)
C
Cumulative fraction
miR-203 targets
Non-targets
P-value=0.118
Log2(Eff)
D
RNA
Eff
0%
50%
100%
(Targets / Non-targets)BAT > (Targets / Non-targets)WAT
(Targets / Non-targets)BAT < (Targets / Non-targets)WAT

Figure S5

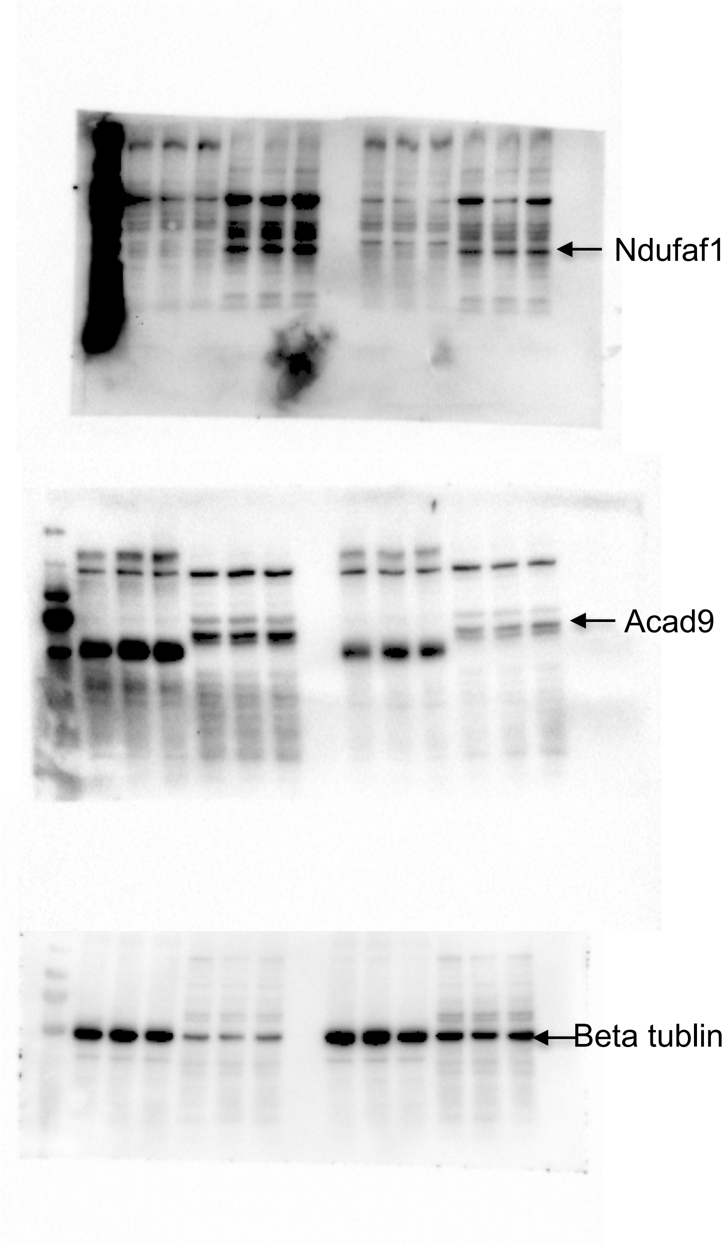

Figure S6
